# MAP kinase p38 is a novel target of CacyBP/SIP phosphatase

**DOI:** 10.1007/s00726-017-2404-7

**Published:** 2017-03-10

**Authors:** Agnieszka M. Topolska-Woś, Sara Rosińska, Anna Filipek

**Affiliations:** Laboratory of Calcium Binding Proteins, Department of Molecular and Cellular Neurobiology, Nencki Institute of Experimental Biology, Polish Academy of Sciences, 3 Pasteur Street, 02-093 Warsaw, Poland

**Keywords:** CacyBP/SIP, ERK1/2, MAPK, NB2a, Oxidative stress, p38 kinase

## Abstract

Mitogen-activated protein (MAP) kinases are important players in cellular signaling pathways. Recently, it has been shown that CacyBP/SIP serves as a phosphatase for one of the MAP kinases, ERK1/2. Through dephosphorylation of this kinase CacyBP/SIP modulates the transcriptional activity of Elk-1 and the activity of the CREB-BDNF pathway. In this work, using NB2a cell lysate and recombinant proteins, we show that CacyBP/SIP binds and dephosphorylates another member of the MAP kinase family, p38. Analysis of recombinant full-length CacyBP/SIP and its three major domains, N-terminal, middle CS and C-terminal SGS, indicates that the middle CS domain is responsible for p38 dephosphorylation. Moreover, we show that CacyBP/SIP might be implicated in response to oxidative stress. Dephosphorylation of phospho-p38 by CacyBP/SIP in NB2a cells treated with hydrogen peroxide is much more effective than in control ones. In conclusion, involvement of CacyBP/SIP in the regulation of p38 kinase activity, in addition to that of ERK1/2, might point to the function of CacyBP/SIP in pro-survival and pro-apoptotic pathways.

**Electronic supplementary material:**

The online version of this article (doi:10.1007/s00726-017-2404-7) contains supplementary material, which is available to authorized users.

## Introduction

CacyBP/SIP (Calcyclin binding protein/Siah-1 interacting protein) initially identified in Ehrlich ascites tumor cells was later found in various mammalian cells and tissues (Filipek and Kuźnicki 1998; Zhai et al. 2008). The role of CacyBP/SIP, although not fully understood, seems to be connected with protein dephosphorylation, ubiquitination, cytoskeletal dynamics, regulation of gene expression, cell proliferation and differentiation, and others (Topolska-Woś et al. 2016). Possible involvement of CacyBP/SIP in many cancers, including gastric, breast, pancreatic, colorectal, brain and renal has also been proposed (Schneider and Filipek 2011; Ning et al. 2016). Recent results indicate that the role of CacyBP/SIP in signaling pathways involved in development of these cancers might be associated with dephosphorylation of a MAP kinase family member, ERK1/2 (Kilanczyk et al. 2011, 2012), followed by changes in the activity of Elk-1 and CREB-BDNF (Kilanczyk et al. 2009, 2015; Rosińska et al. 2016) and/or with participation in cellular response to oxidative stress (Topolska-Woś et al. 2015).

The mammalian p38 kinase family is composed of four isoforms: p38α, p38β, p38γ and p38δ (Saba-El-Leil et al. 2016). Among these isoforms, p38α and p38β are the most closely related (Cuenda and Rousseau 2007). These two isoforms are ubiquitously expressed, although p38α is the predominant one in most tissues. Gene disruption studies have revealed that loss of p38α is lethal due to defects in placenta morphogenesis (Adams et al. 2000; Allen et al. 2000; Mudgett et al. 2000; Tamura et al. 2000). p38α is activated by a wide variety of environmental stresses or inflammatory cytokines and is involved in orchestrating cellular response to different signals. Activation of p38α by oxidative stress, in fact by reactive oxygen species (ROS), plays an important role in mediating cell death (Cai et al. 2006; Harada et al. 2014). For example, ROS-induced p38 activation was shown to inhibit tumorigenesis (Gutiérrez-Uzquiza et al. 2012) or to play a role in the pathology of Alzheimer’s disease. In the latter case, it has been reported that p38 kinase co-immunoprecipitated with paired helical filaments (Zhu et al. 2000).

Since p38 kinase is activated by stress and given the phosphatase activity of CacyBP/SIP towards ERK1/2 and its involvement in cellular response to oxidative stress (Topolska-Woś et al. 2015), in this work we examined the interaction of CacyBP/SIP with p38 MAP kinase and the influence of CacyBP/SIP on p38 phosphorylation status.

## Materials and methods

### Culture of NB2a cells

Mouse neuroblastoma NB2a cells at passages 15–25 were grown in EMEM containing 10% FBS (Life Technologies, USA), 25 mM sodium bicarbonate, penicillin 100 μg/ml, streptomycin 100 μg/ml, 2 mM l-glutamine and fungizone 0.25 μg/ml. Media were changed every 2–3 days and cells were passaged when confluent. Prior to all experiments NB2a cells were differentiated using palmitoyl-l-carnitine (Topolska-Woś et al. 2015). Cells, at 50% confluence, were grown for 24 h in a medium with decreased FBS concentration (5%) and supplemented with palmitoyl-l-carnitine at a final concentration of 100 µM.

### Cell transfection, oxidative stress and preparation of protein lysates

NB2a cells (70–80% confluent) were transfected with the expression plasmids: p3xFLAG-CMV-10-CacyBP/SIP or p3xFLAG-CMV-10, as a control, using Lipofectamine 2000 (Invitrogen, Thermo Fisher Scientific, USA) according to the manufacturer’s protocol. After 24 h cells were harvested in order to prepare cell lysates.

To induce oxidative stress, transfected NB2a cells were treated with 0.004% hydrogen peroxide (H_2_O_2_) or left untreated (control). In both cases a medium devoid of antibiotics was used and cells were harvested after 3 h.

Total protein lysates were obtained by suspending cells in a buffer containing: 20 mM Tris pH 7.5, 50 mM KCl, 3 mM MgCl_2_, 0.1% Triton X-100 and protease inhibitors (Complete Mini EDTA-free; Roche, Switzerland). Cells were homogenized mechanically by running 30 times through a gauge needle (MYJECTOR U-40 insulin, Terumo, Germany). Afterwards, the sample was centrifuged at 10,000×*g* for 10 min at 4 °C. Protein concentration in the supernatant (lysate) was estimated by the Bradford assay (Bio-Rad, USA) and samples containing 50 μg of protein were analyzed by SDS–PAGE and western blot.

### Proximity ligation assay

For visualization of CacyBP/SIP-p38 complex the proximity ligation assay (PLA) was employed. NB2a cells, grown on coverslips pretreated with 50 μg/ml poly-l-lysine (Sigma-Aldrich, Germany), were fixed with 3% paraformaldehyde in PBS for 20 min at room temperature. Then, the coverslips were washed with PBS, incubated for 10 min at room temperature with 50 mM NH_4_Cl in PBS and washed with PBS. Cells were permeabilized with 0.1% Triton X-100 in PBS for 5 min at 4 °C and then washed again in PBS. For blocking, coverslips were incubated with 5% FBS in PBS at room temperature for 1 h. The reaction with primary antibodies, rabbit polyclonal anti-CacyBP/SIP (#3354, Cell Signaling, USA) diluted 1:50 and mouse monoclonal anti-phospho-p38 diluted 1:50 (#9211, Cell Signaling, USA) was carried out for 1.5 h at 37 °C. After washing, the incubation with anti-rabbit PLA plus and anti-mouse PLA minus probes (1:4) was conducted for 2 h at 37 °C in a humidity chamber. All following steps were performed according to the manufacturer’s protocol and with the reagents and media provided in the PLA-kit (Sigma-Aldrich, Germany).

### Plasmids, protein expression and purification

Plasmids, pET28-CacyBP/SIP_FL_, pET28-CacyBP/SIP_1–77_, pET28-CacyBP/SIP_74–178_, pET28-CacyBP/SIP_178–229_ and pGEX-4T1-p38α for bacteria transformation were prepared as described previously (Topolska-Woś et al. 2015; Filipek et al. 2002; Ge et al. 2002). His_6_-tagged CacyBP/SIP and p38α-GST proteins were expressed in *E. coli* BL21 strain and purified by gravity flow using TALON^®^ Co^2+^ metal affinity (Clontech, Takara Bio, USA) and Glutathione-Sepharose 4B (Amersham Biosciences, UK) resins, respectively. The GST tag was removed from p38α using 0.2 U of thrombin (Novagen, Merck Millipore, Germany) per 1 mg of protein during overnight incubation at 20 °C, according to the manufacturer’s protocol. Proteins, full-length CacyBP/SIP and its domains as well as p38α, were dialyzed against buffer containing 150 mM NaCl, 20 mM Tris–HCl, pH 8.4 and then concentrated using centrifugal filters (Amicon, Millipore Corporation, USA).

### SDS–PAGE and western blot

Gel electrophoresis with 12% (w/v) polyacrylamide containing 0.1% SDS was performed by the method of Laemmli (1970). Proteins were transferred electrophoretically onto nitrocellulose and identified using rabbit polyclonal antibodies: anti-CacyBP/SIP, anti-phospho-Thr180/Tyr182-p38, anti-p38α (#9212, Cell Signaling, USA) diluted 1:1000 or mouse anti-β-actin HRP-linked monoclonal antibody (#A3854, Sigma-Aldrich, Germany) diluted 1:40,000. After washing with TBS-T buffer (50 mM Tris pH 7.5, 200 mM NaCl, 0.05% Tween 20) blots were allowed to react with secondary goat anti-rabbit IgG antibody (#NA934V, GE Healthcare, UK) diluted 1:10,000, conjugated to horseradish peroxidase. After three washes with TBS-T and two washes with TBS (50 mM Tris pH 7.5, 200 mM NaCl) blots were developed with the ECL chemiluminescence kit (Bio-Rad, USA), followed by exposure against Kodak X-ray film. The intensity of the protein bands was quantified using an Ingenius instrument (Syngene, Poland) and analyzed using the ImageJ software (NIH, USA) with β-actin as a reference protein for the total protein lysate.

## 2D electrophoresis

200 µg of protein from the cell lysate of neuroblastoma NB2a cells, precipitated with TCA and washed with cold acetone, were suspended in a loading buffer containing 8 M urea, 2% CHAPS, 50 mM DTT, 0.2% ampholine (Bio-Lyte pH 3–10, Bio-Rad, USA). Samples were applied on a linear 7-cm-long pH gradient (3–10) strips (Bio-Rad, USA). After 1 h of incubation at room temperature, strips were covered with mineral oil (Bio-Rad, USA) and incubated at room temperature for 16 h. After a hydration step strips were applied into the Protean IEF Cell instrument (Bio-Rad, USA) and iso-electrofocusing was carried out for 20 h with the maximal voltage of 10 kVh. The final voltage was 4000 V. Strips were then washed for 30 min in buffer containing 375 mM Tris–HCl pH 8.8, 6 M urea, 2% SDS, 20% glycerol and 2% DTT. Washing was performed for the next 30 min in buffer containing 2% iodocetamide instead of DTT. Incubation was followed by standard SDS–PAGE (12% polyacrylamide gel) and western blot analysis.

### ELISA

Recombinant p38α and BSA (Serva, Germany) were coated on a 96-well plate (400 ng/well) in the Kinase Buffer (Cell Signaling, USA) and incubated overnight at 4 °C. After incubation wells were washed three times with PBS-T (PBS containing 0.05% Tween 20). The remaining adsorption sites were blocked at room temperature for 2 h with PBS-T supplemented with 3% BSA. After washing, increasing amounts of recombinant CacyBP/SIP were added in 50 μl of the reaction buffer (50 mM KCl, 25 mM HEPES pH 7.6, 2 mM DTT, 0.05% Triton X-100, 5 mM MgCl_2_, 5% glycerol). After 1.5 h incubation at room temperature, wells were washed and primary rabbit polyclonal anti-CacyBP/SIP antibody (Cell Signaling, USA) diluted 1:3000 in PBS-T containing 3% BSA was added. After overnight incubation and washing the secondary anti-rabbit antibody diluted 1:10,000 in the same buffer, was applied. After 1-h incubation and washing the colorimetric detection with the TMB peroxidase EIA substrate kit (Bio-Rad, USA) was performed. The reaction was stopped with 1 M sulfuric acid and the absorbance was measured at 450 nm using a microplate reader (Thermo Labsystems, Thermo Fisher Scientific, USA).

### In vitro phosphatase activity assay

Recombinant p38α was autophosphorylated for 30 min at 30 °C in the presence of the Kinase Buffer supplemented with 0.2 mM ATP. In order to remove the excess of ATP and phosphatase inhibitors from the Kinase Buffer, samples were diluted and concentrated to the starting volume using centrifugal filters (Amicon, Millipore Corporation, USA). Dephosphorylation of phospho-p38α by recombinant full-length CacyBP/SIP or its domains: N-terminal (1–77), middle CS (74–178) and C-terminal (178–229), at a 1:1 molar ratio, was carried out for 40 min at 37 °C. Aliquots containing 2 µg of p38α were collected every 10 min and the reaction was stopped by addition of SDS–PAGE sample buffer. Afterwards, proteins were separated by SDS–PAGE and analyzed by western blot using anti-phospho-p38 antibody.

### Statistical analysis

The results of western blots are presented as mean ± SEM of at least three independent experiments. Differences in mean values were tested by ANOVA, post hoc and multicomparison test. The level of statistical significance was set either at *p ≤ 0.05 or **p ≤ 0.01.

## Results

### Interaction with and dephosphorylation of phospho-p38 by CacyBP/SIP in neuroblastoma NB2a cells

**Fig. 1:**
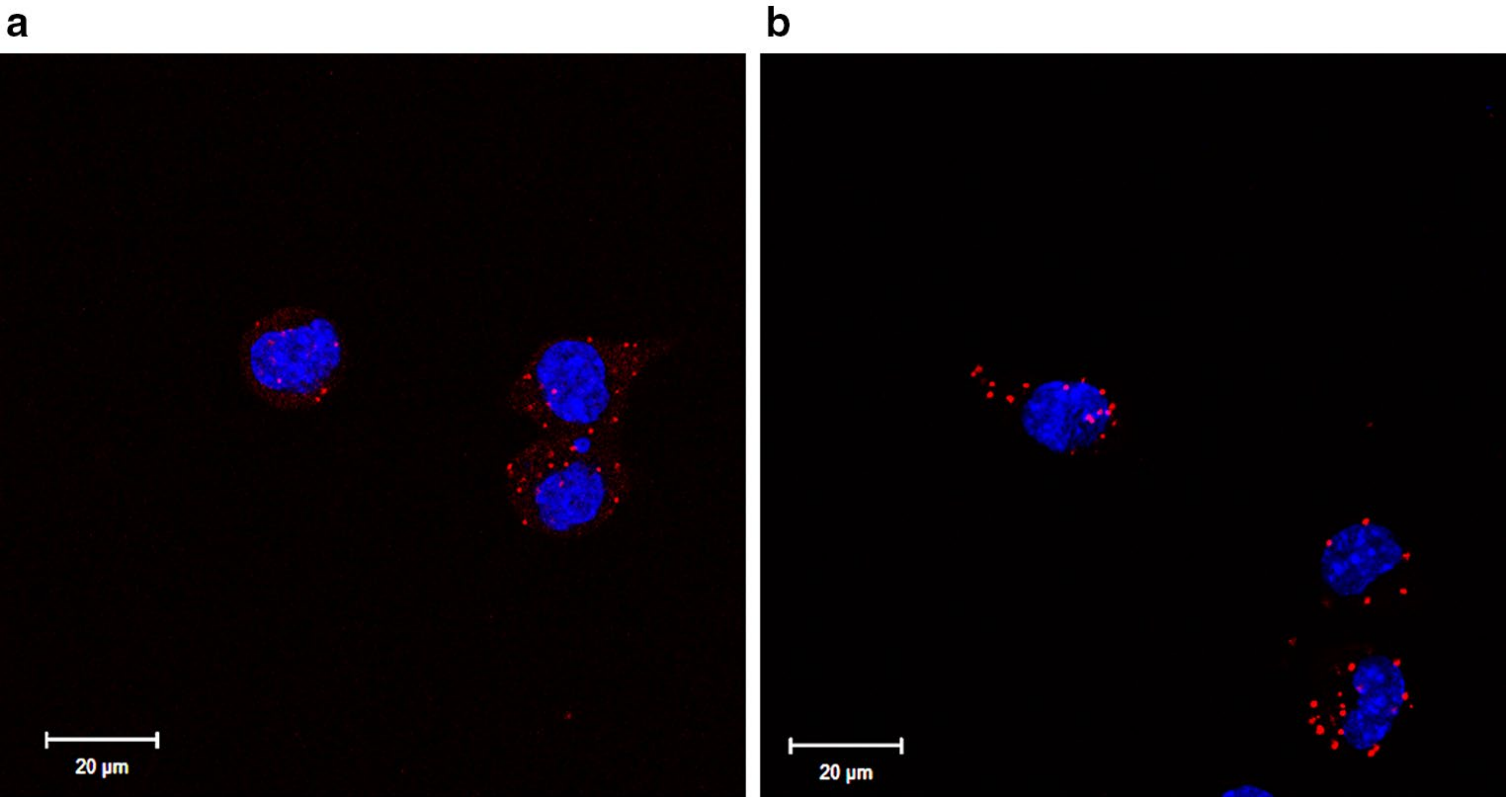
Presence of phospho-p38-CacyBP/SIP complexes in NB2a cells visualized by the PLA assay. **a** Images of control cells. **b** Images of cells treated with hydrogen peroxide (H_2_O_2_). Complexes of examined proteins are in *red*; cell nuclei, stained with DAPI, are in *blue*. *Scale bar* is 20 μm (color figure online)

To check whether CacyBP/SIP forms a complex with p38 kinase in NB2a cells, we performed the proximity ligation assay (PLA) with the use of anti-CacyBP/SIP and anti-phospho-Thr180/Tyr182-p38 antibodies. The results of this experiment showed that CacyBP/SIP interacts with the phosphorylated form of p38 kinase in the cytoplasm of NB2a cells, as indicated by red PLA signals (Fig. 1a). Since it is known that oxidative stress causes an increase in p38 phosphorylation/activity (Gutiérrez-Uzquiza et al. 2012), in our studies NB2a cells were exposed to hydrogen peroxide (H_2_O_2_). After H_2_O_2_ treatment the number of PLA signals was higher, as compared to control, untreated cells (Fig. 1b). For the appropriate control of interaction specificity NB2a cells were incubated with both primary antibodies but without ligase, a critical reagent in the PLA assay. As it is shown in Supplementary Fig. 1, no signals were detected in that case.

**Fig. 2:**
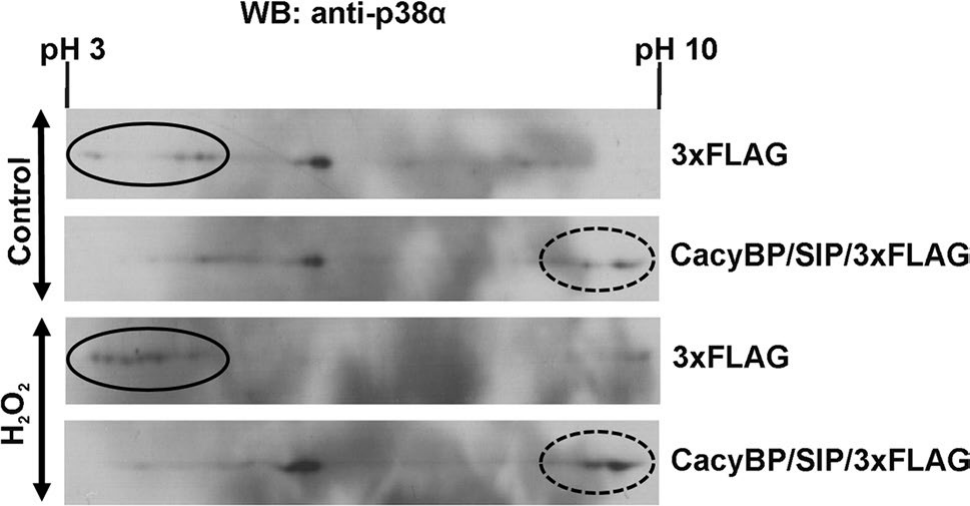
p38α forms with different pI values in NB2a cell lysate. Lysates obtained from cells transfected with plasmids encoding 3xFLAG or CacyBP/SIP-3xFLAG were analyzed by 2D electrophoresis. *Two upper panels* show the pattern of p38α spots in lysates from untreated cells (control) while *two lower panels* show lysates from cells treated with hydrogen peroxide (H_2_O_2_). Representative western blots, out of 3 performed, are shown

It has already been established that CacyBP/SIP exhibits phosphatase activity towards ERK1/2 kinase (Kilanczyk et al. 2011). This result, together with the fact that CacyBP/SIP co-localizes with phospho-p38 kinase, prompted us to examine whether CacyBP/SIP might dephosphorylate this kinase in neuroblastoma NB2a cells. To check this hypothesis, lysates obtained from cells overexpressing CacyBP/SIP with a 3xFLAG tag or the 3xFLAG alone were subjected to 2D electrophoresis. As it can be seen in Fig. 2, the pattern of the p38α forms in control and H_2_O_2_-treated NB2a cells was changed due to increased level of CacyBP/SIP. A shift in the direction of higher pI (compare spots marked by circles versus those marked by dashed circles) is visible which indicates that CacyBP/SIP might dephosphorylate phospho-p38α kinase.

**Fig. 3:**
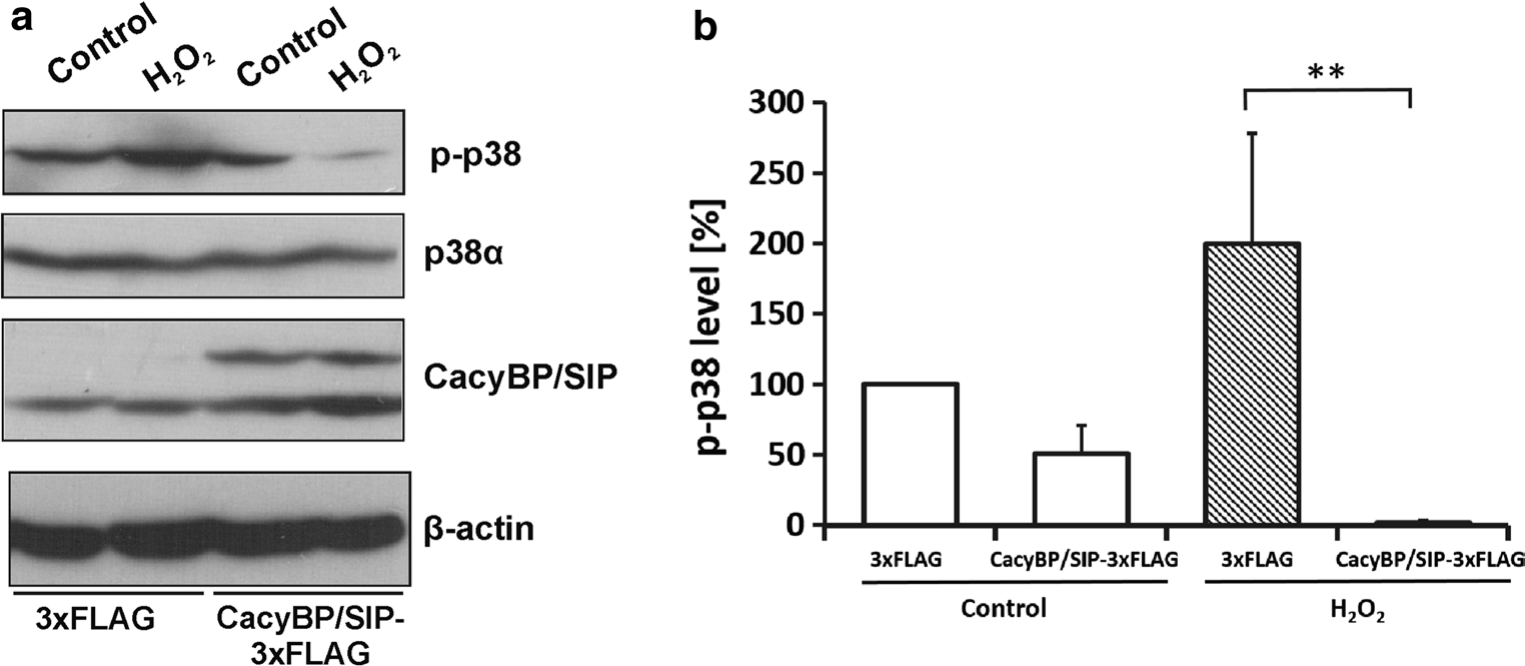
Level of phospho-p38 in NB2a cells overexpressing CacyBP/SIP. Cells overexpressing CacyBP/SIP-3xFLAG or 3xFLAG alone were left untreated (control) or were treated with hydrogen peroxide (H_2_O_2_). Cell lysates (50 µg protein) were subjected to SDS–PAGE and western blot developed with anti-phospho-p38, anti-p38α, anti-CacyBP/SIP or anti-β-actin antibodies. Staining with anti-β actin antibody shows that each lane contains a similar amount of protein. **a** Representative western blot, out of 3 performed, and **b** densitometric analysis of western blot results (mean ± SEM; **p ≤ 0.01). *White bars* show phospho-p38 (p-p38) level in untreated cells (control) while *grey bars* show phospho-p38 (p-p38) level in H_2_O_2_-treated NB2a cells

To confirm the results of PLA and 2D assays, we performed SDS–PAGE and western blot with anti-phospho-p38 antibody using lysates prepared from control or CacyBP/SIP overexpressing NB2a cells treated or not with H_2_O_2_. As it can be seen in Fig. 3, overexpression of CacyBP/SIP causes a decrease in the phospho-p38 level. Interestingly, the effect of CacyBP/SIP was much stronger when cells were subjected to oxidative stress. Densitometric analysis of the results obtained from western blots revealed that the level of phospho-p38 in H_2_O_2_-treated cells was very low (Fig. 3b).

### In vitro binding and dephosphorylation of phospho-p38α by CacyBP/SIP

**Fig. 4:**
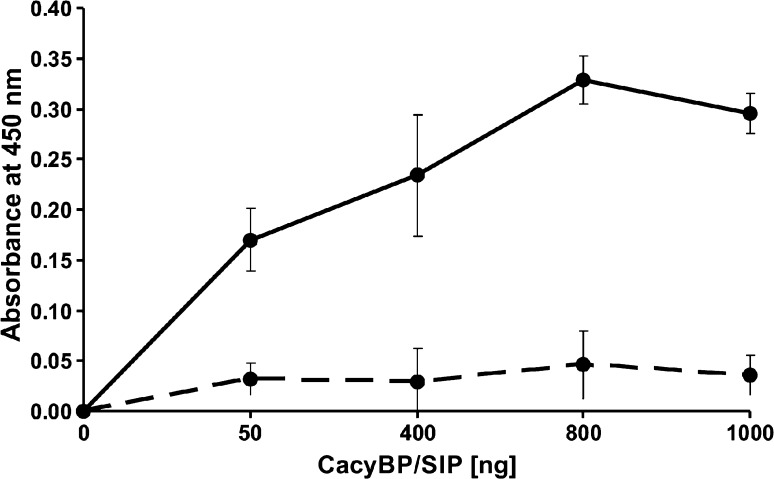
Binding of purified recombinant CacyBP/SIP and p38α analyzed by ELISA. The plate was coated with p38α or BSA (control) and then increasing amounts of CacyBP/SIP were applied. After incubation with anti-CacyBP/SIP antibody colorimetric detection was performed. Results are presented as mean ± SD. *Dashed line* control, *solid line* CacyBP/SIP

To check whether the purified recombinant CacyBP/SIP and p38α might bind to each other we performed an ELISA assay. As can be seen in Fig. 4, an increase in absorption values was observed in the presence of CacyBP/SIP and p38α, and not in the presence of BSA and p38α. This suggests that the interaction between CacyBP/SIP and p38α is direct.

**Fig. 5:**
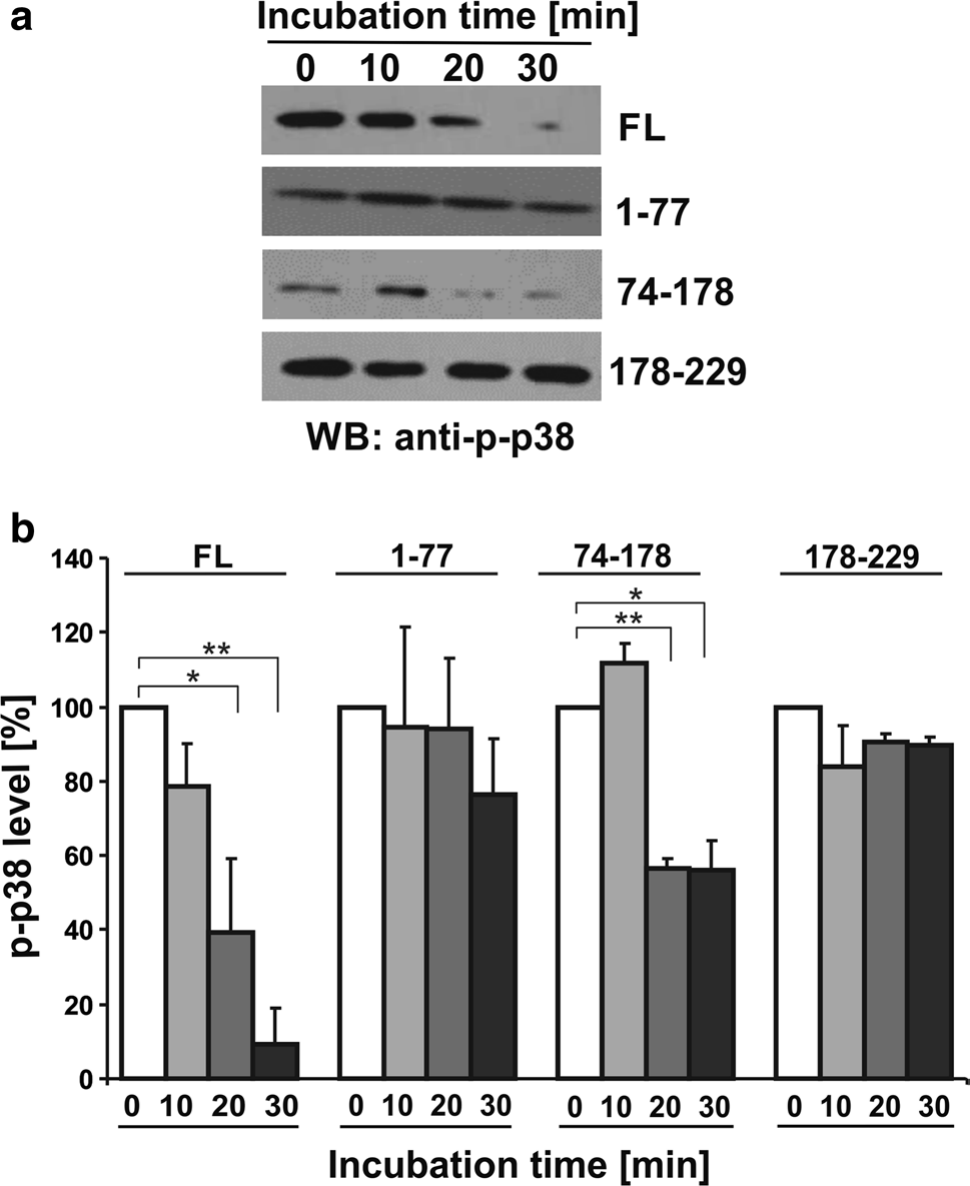
Level of phospho-p38α (p-p38α) after incubation with CacyBP/SIP or its domains—an in vitro assay. Purified recombinant full-length CacyBP/SIP (FL) or its domains: N-terminal (1–77), middle CS (74–178) and C-terminal (178–229) were incubated with recombinant autophosphorylated p38α kinase for various times. **a** Representative western blot, out of 3 performed and **b** densitometric analysis of western blot results (mean ± SEM; **p ≤ 0.01 and *p ≤ 0.05)

**Fig. 6:**
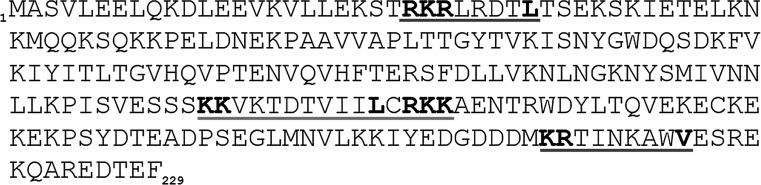
In silico analysis of the mouse CacyBP/SIP amino acid sequence. *Blue lines* indicate KIM motifs responsible for ERK1/2 binding in the N- and C-terminal domains, while *red line* indicates a putative motif for p38 binding in the middle CS domain of CacyBP/SIP, respectively (color figure online)

To establish whether CacyBP/SIP dephosphorylates p38α under in vitro conditions, the kinase was autophosphorylated and then CacyBP/SIP was added. Taking into account that the N- and C-terminal domains of CacyBP/SIP were shown to be involved in ERK1/2 dephosphorylation, we have analyzed full-length CacyBP/SIP and its three major domains. The results presented in Fig. 5 show that in the presence of CacyBP/SIP the level of phospho-Thr180/Tyr182 p38α was diminished. Moreover, it seems that the middle CS domain is responsible for phospho-p38α dephosphorylation. As it can be seen in this figure, in the case of the N- and C-terminal domains the differences were not statistically significant. Thus, in the next step, we performed in silico analysis to search for a potential p38-binding site within the mouse CacyBP/SIP amino acid sequence. Interestingly, besides the two KIM motifs identified and reported earlier for ERK1/2 kinase (Topolska-Woś et al. 2015), a motif for p38 binding in the middle CS domain was identified (Fig. 6). The presence of a p38 binding motif in the middle CS domain of CacyBP/SIP is in agreement with the experimental results showing dephosphorylation of phospho-p38α kinase by this domain.

## Discussion

The p38 MAPK pathway, strongly activated by stress, plays an important role in the regulation of cell survival and differentiation as well as in the immune response (Ono and Han 2000; Nebreda and Porras 2000; Cuenda and Rousseau 2007). Studies using gene-targeting in mice have provided important information regarding regulation of p38α activity and its function in vivo. It has been shown that p38α might be implicated in maintaining tissue homoeostasis and in several pathologies including inflammation, cancer, heart diseases and neurodegenerative diseases (Cuenda and Rousseau 2007).

Activation of p38 kinase requires phosphorylation that induces conformational changes facilitating substrate binding. p38α is mainly phosphorylated by MKK3, MKK6 and MKK4. The relative contribution of different kinases to p38*α* activation depends on the stress/stimulus and also on the cell type. As for other kinases, not only activation but also deactivation plays an important role, both result in specific outcomes. Termination of p38 kinase activity burst involves several phosphatases (Zhang et al. 2008) including those that dephosphorylate serine/threonine residues, such as PP2A and PP2C, or those that dephosphorylate tyrosine residues, such as STEP, HePTP and PTP-SL. The activity of MAPKs can also be regulated by a family of DUSPs (dual-specificity phosphatases), which dephosphorylate both tyrosine and threonine residues (Cuadrado and Nebreda 2010).

Because CacyBP/SIP was shown to serve as ERK1/2 phosphatase, in this work we examined the interaction with and dephosphorylation of p38 kinase by CacyBP/SIP. We reveal that CacyBP/SIP dephosphorylates this kinase in NB2a cell lysate and in vitro. Since it is known that p38 kinase is activated by oxidative stress, in our studies NB2a cells were exposed to H_2_O_2_. We found that in this case binding and dephosphorylation of this kinase by CacyBP/SIP was much more effective. The exact residue-specificity or dual-specificity of CacyBP/SIP phosphatase was not defined, although phospho-p38 is likely dephosphorylated on the residues at the activation loop, associated with the main activity of this kinase. Using the in vitro assay we show that the middle CS domain might be responsible for phospho-p38 dephosphorylation. In agreement with these data are the results of in silico analysis indicating a potential p38-binding motif within the middle domain of CacyBP/SIP. The motif comprised KK–X_7-8_-I/L_2_-(K/R)_3-5_ template, where X is any amino acid, similar to those found in other p38-specific phosphatases (Liu et al. 2016). Moreover, p38-binding motif overlaps a standard bipartite nuclear localization sequence (NLS). Thus, in can be speculated that interaction of p38 with CacyBP/SIP might have an effect on CacyBP/SIP cellular localization (Roux and Blenis 2004).

CacyBP/SIP is a ubiquitously expressed protein. Its high level is observed in brain, spleen, thymus and in many cancers (Schneider and Filipek 2011; Ning et al. 2016). On the basis of published data (Kilanczyk et al. 2009, 2011, 2012) and present results, it can be suggested that the role of CacyBP/SIP in signaling pathways engaged in development of cancers might be associated with dephosphorylation of MAP kinases and/or with its involvement in cellular response to oxidative stress (Topolska-Woś et al. 2015). In conclusion, contribution of CacyBP/SIP phosphatase to the regulation of p38 and ERK1/2 kinases makes it an important player in signaling pathways leading to apoptosis or cell survival.

## Electronic supplementary material

Below is the link to the electronic supplementary material.

Supplementary material 1 (TIFF 5410 kb) **Supplementary Fig. 1 PLA assay. A** Images of control and **B** hydrogen peroxide treated NB2a cells processed without incubation with ligase. Cell nuclei, stained with DAPI, are in *blue*. Scale bar is 20 μm

## Abbreviations

BSA: Bovine serum albumin
CacyBP/SIP: Calcyclin binding protein/Siah-1 interacting protein
DTT: Dithiothreitol
FBS: Fetal bovine serum
MAPK: Mitogen-activated protein kinase
PBS: Phosphate buffered saline
PBS-T: PBS containing 0.05% Tween 20
ROS: Reactive oxygen species
TCA: Trichloroacetic acid
