# Supplementary figures and images for: MAP kinase p38 is a novel target of CacyBP/SIP phosphatase
Source: Amino Acids. 2017 Mar 10;49(6):1069–76. doi: 10.1007/s00726-017-2404-7 (PMC5437258; doi:10.1007/s00726-017-2404-7)

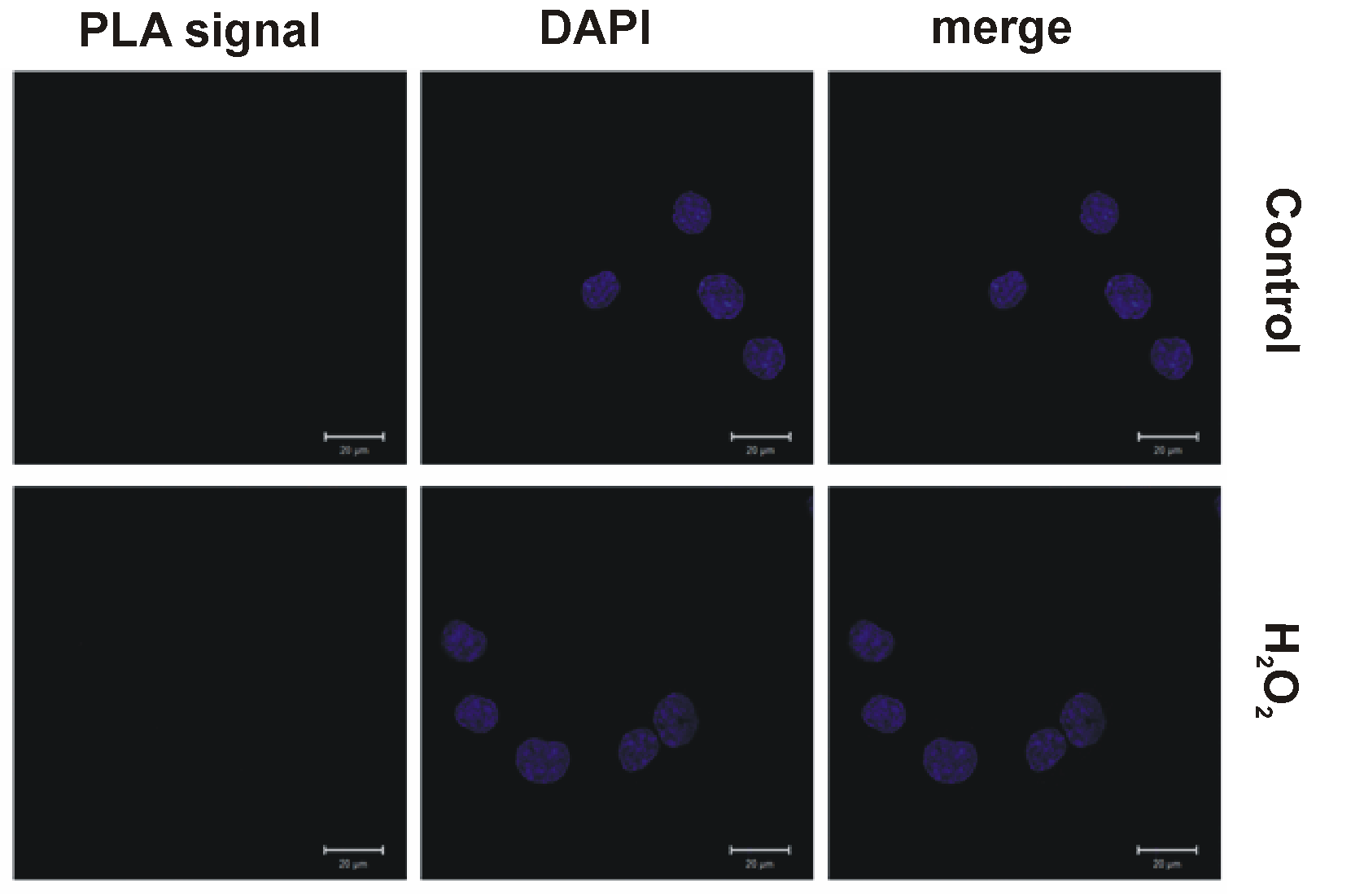

Supplement: Supplementary file 1 — Supplementary material 1 (TIFF 5410 kb) Supplementary Fig. 1 PLA assay. A Images of control and B hydrogen peroxide treated NB2a cells processed without incubation with ligase. Cell nuclei, stained with DAPI, are in blue. Scale bar is 20 μm [file 726_2017_2404_MOESM1_ESM.tif]
